# Knockdown of miR-27a sensitizes colorectal cancer stem cells to TRAIL by promoting the formation of Apaf-1-caspase-9 complex

**DOI:** 10.18632/oncotarget.16779

**Published:** 2017-04-01

**Authors:** Rui Zhang, Jian Xu, Jian Zhao, Jinghui Bai

**Affiliations:** ^1^ Department of Colorectal Surgery, Cancer Hospital of China Medical University, Liaoning Cancer Hospital and Insititute, Shenyang 110042, China; ^2^ Department of Internal Medicine, Cancer Hospital of China Medical University, Liaoning Cancer Hospital and Insititute, Shenyang 110042, China

**Keywords:** colorectal cancer stem cells, miR-27a, TRAIL, Apaf-1, caspase-9

## Abstract

MicroRNAs have been proved to participate in multiple biological processes in cancers. For developing resistance to cytotoxic drug, cancer cells, especially the cancer stem cells, usually change their microRNA expression profile to survive in hostile environments. In the present study, we found that expression of microRNA-27a was increased in colorectal cancer stem cells. High level of microRNA-27a was indicated to induce the resistance to TNF-related apoptosis-inducing ligand (TRAIL). Knockdown of microRNA-27a resensitized colorectal cancer stem cells to TRAIL-induced cell death. Mechanically, the gene of Apaf-1, which is associated with the mitochondrial apoptosis, was demonstrated to be the target of microRNA-27a in colorectal cancer stem cells. Knockdown of microRNA-27a increased the expression level of Apaf-1, thus enhancing the formation of Apaf-1-caspase-9 complex and subsequently promoting the TRAIL-induced apoptosis in colorectal cancer stem cells. These findings suggested that knockdown of microRNA-27a in colorectal cancer stem cells by the specific antioligonucleotides was potential to reverse the chemoresistance to TRAIL. It may represent a novel therapeutic strategy for treating the colorectal cancer more effectively.

## INTRODUCTION

Colorectal cancer represents the third most common cancer around the world [1]. Because of the liver metastases in early stage, colorectal cancer is realized as the “leading killer”, which has become a serious threat to human life and health [2, 3]. For treatment of colorectal cancer, chemotherapy is efficient initially. However, as the continuous use of anti-tumor drugs, colorectal cancer cells show lower response to them gradually [4]. Recently, studies demonstrate that cancer stem cells (CSCs) are responsible for drug-resistance in various cancers [5–7], which emphasizes the significance of targeting CSCs in cancer therapy. CSCs are highly self-renewing and tumorigenic cells in tumor. They show obvious chemoresistance and play key roles in chemotherapy failure and cancer relapse [8, 9]. Previous studies have identified that CD133 on cell surface is the marker of colorectal CSCs [10]. It is urgent to explore the mechanism by which colorectal CSCs develop their resistance to anti-tumor drugs for improving the efficiency of cancer therapy.

In TNF superfamily, the cytokine of TNF-related apoptosis-inducing ligand (TRAIL) is considered as a potential anti-tumor agent. Treatment with TRAIL effectively kills multiple cancer cells, such as hepatocellular carcinoma cells and colorectal cancer [11, 12]. As TRAIL induces significant apoptosis selectively in tumor cells without influencing the function of normal cells, it has been tested in clinical trials for treatment of various cancers [13]. However, CSCs, which are the special population in cancers, are reported to be resistant to TRAIL treatment [14]. It is significant to overcome this obstacle in TRAIL therapy by targeting the colorectal CSCs.

MicroRNAs are small and non-coding RNAs exist in cells. They are endogenous and involved in regulating the expression of human genes. MicroRNAs induce degradation of targeting mRNA by pairing with partially complementary sequence in the 3′-untranslated region [15]. As microRNAs regulate about 60% of the human genes, they participate in various biological processes in cells [16, 17]. In cancer, microRNAs are often dysregulated. Correcting the dysregulation of microRNAs has been reported to inhibit cell proliferation, metastasis, survival and chemoresistance of cancers [18–20]. In the present study, we found that expression of microRNA-27a (miR-27a) was increased in colorectal CSCs rather than their corresponding colorectal cells. Moreover, knockdown of miR-27a was found to sensitize the colorectal CSCs to TRAIL treatment. We aim to explore the molecular mechanism of miR-27a antioligonucleotides on promoting the TRAIL-induced apoptosis in colorectal CSCs.

## RESULTS

### Expression level of miR-27a is increased in colorectal cancer stem cells

**Figure 1:**
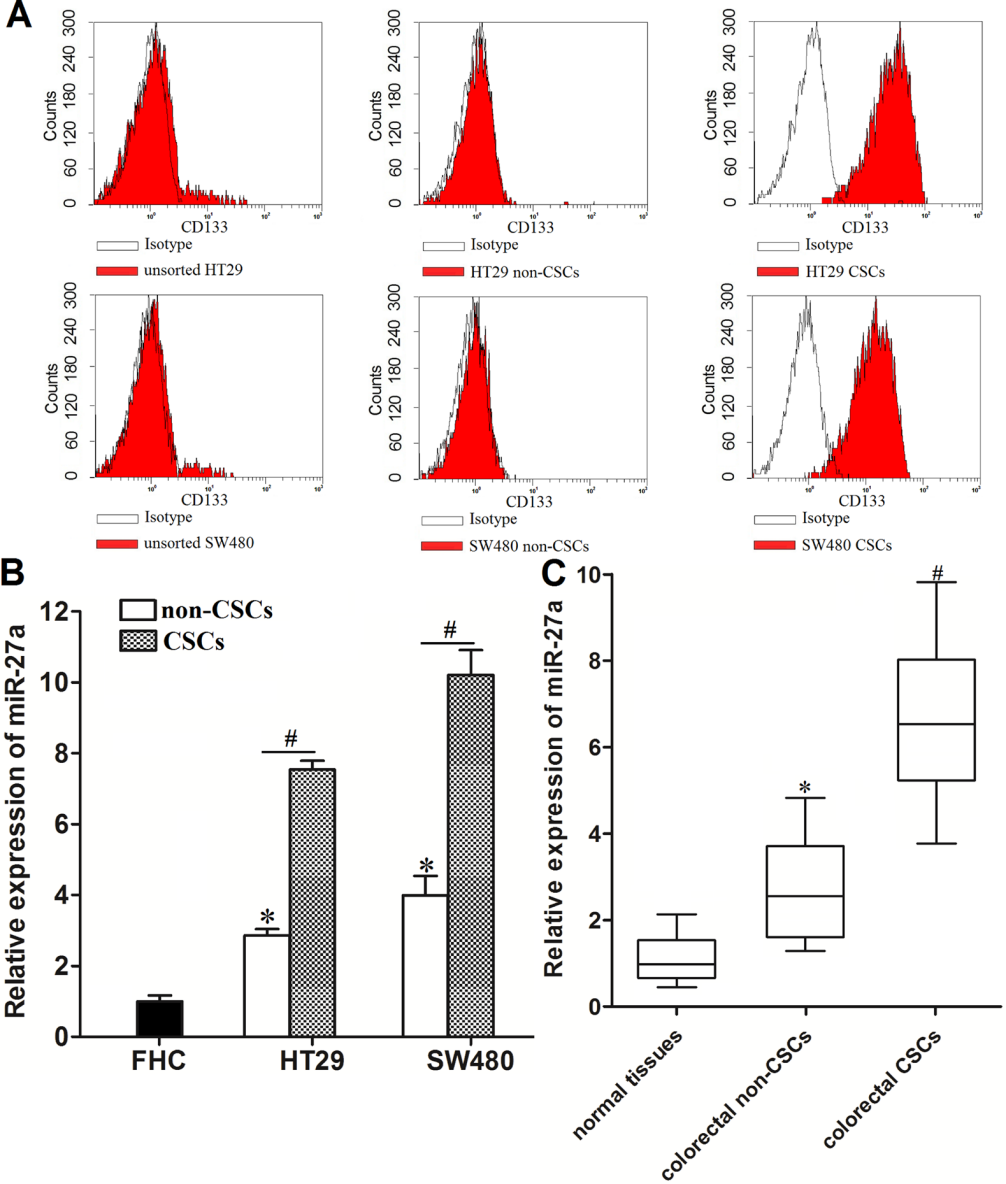
Overexpresion of miR-27a in colorectal cancer stem cells (**A**) Flow cytometry analysis was performed to detect the populations of CSCs and non-CSCs in HT29 and SW48 cell lines. (**B**) Expression of miR-27a in FHC, HT29 and SW480 CSCs and non-CSCs was measured by using qRT-PCR analysis. *P < 0.05 *vs*. FHC cells. ^#^*P* < 0.05. (**C**) Expression of miR-27a in normal tissues, colorectal CSCs and non-CSCs was measured by using qRT-PCR analysis. *P < 0.05 *vs*. normal tissues. ^#^*P* < 0.05 *vs*. colorectal non-CSCs.

To explore the role of miR-27a in colorectal cancer stem cells, we separated the CSCs population from the HT29 and SW480 cell lines by using the CD133 antibody. As shown in Figure 1A, isolated HT29 and SW480 CSCs expressed high level of CD133. On contrary, the expression of CD133 on the surface of HT29 and SW480 non-CSCs was low. Next, we investigated the expression of miR-27a in HT29 and SW480 CSCs and non-CSCs. We observed that miR-27a was overexpressed in colorectal cancer cells rather than the normal colorectal cell line FHC. Moreover, the population of CSCs in HT29 and SW480 colorectal cells expressed significantly higher level of miR-27a compared to their corresponding non-CSCs population (Figure 1B). To validate the overexpression of miR-27a in colorectal CSCs, we collected the CSCs and non-CSCs from the colorectal cancer patients’ tumor tissues. We found that colorectal CSCs showed strongly higher level of miR-27a rather than their corresponding non-CSCs (Figure 1C). Taken together, we demonstrated the increased expression level of miR-27a in colorectal cancer stem cells.

### MiR-27a antioligonucleotides sensitize colorectal cancer stem cells to TRAIL

**Figure 2:**
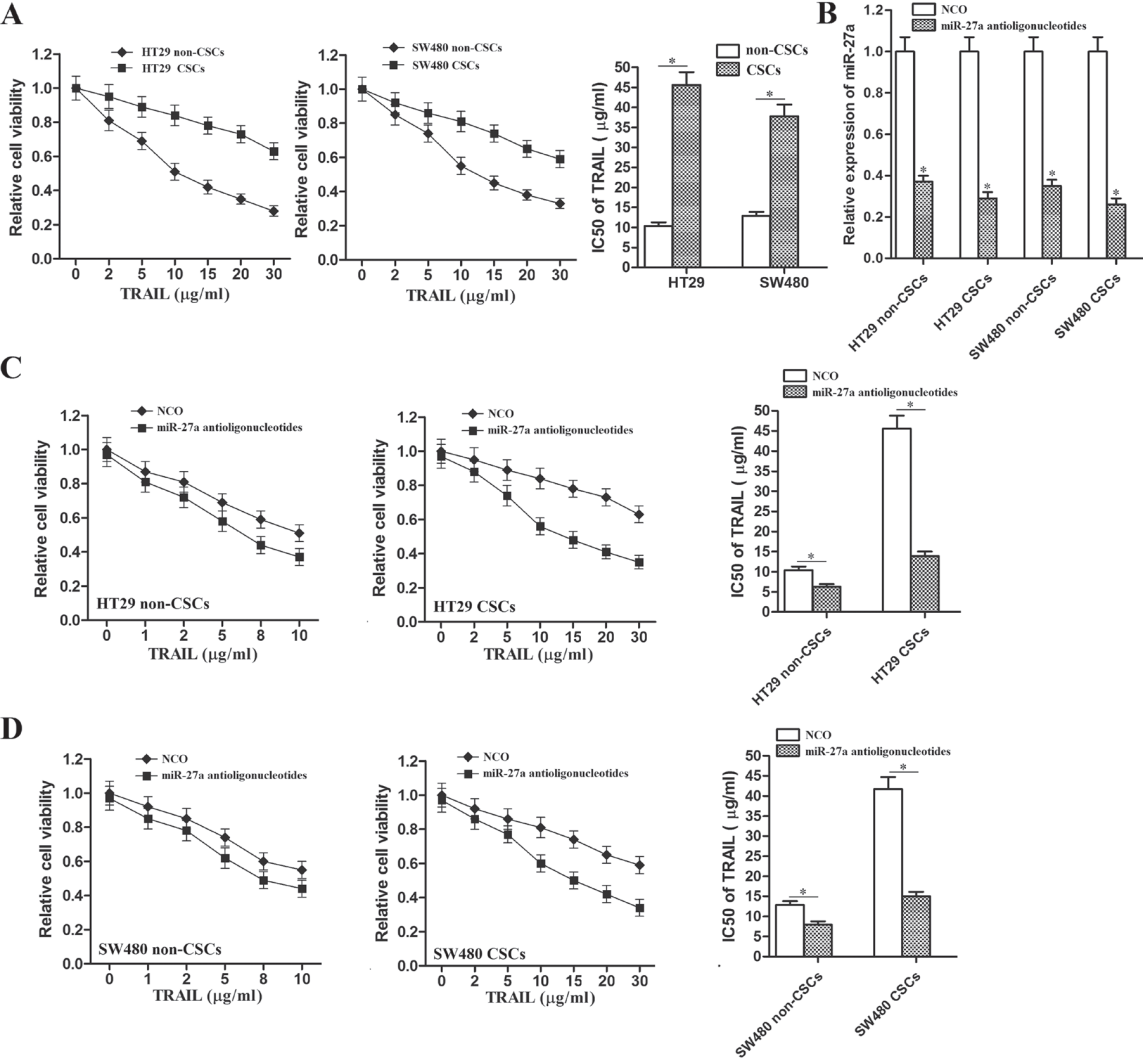
MiR-27a antioligonucleotides sensitized colorectal CSCs to TRAIL (**A**) CCK-8 assays were performed to evaluate the sensitivity of HT29 and SW480 CSCs and non-CSCs to TRAIL. *P < 0.05. (**B**) After transfection with miR-27a antioligonucleotides, relative expression of miR-27a in HT29 non-CSCs, HT29 CSCs, SW480 non-CSCs and SW480 CSCs was detected by qRT-PCR analysis. *P < 0.05 *vs*. NCO group. (**C**) CCK-8 assays were performed to evaluate the effect of miR-27a antioligonucleotides on TRAIL-induced cell death in HT29 CSCs and non-CSCs. *P < 0.05. (**D**) CCK-8 assays were performed to evaluate the effect of miR-27a antioligonucleotides on TRAIL-induced cell death in SW480 CSCs and non-CSCs. *P < 0.05.

We observed that colorectal CSCs exhibited significant resistance to TRAIL treatment. IC50 of TRAIL to HT29 CSCs was 3.38 fold higher than the HT29 non-CSCs, and IC50 of TRAIL to SW480 CSCs was 1.93 fold higher than the SW480 non-CSCs (Figure 2A). The results indicated that colorectal CSCs showed obvious resistance to TRAIL. As the miR-27a was found to be overexpressed in colorectal cancer stem cells, we knockdown the miR-27a by its specific antisense oligonucleotides to investigate the relationship between miR-27a and sensitivity to TRAIL in colorectal cancer (transfection efficiency of miR-27a antioligonucleotides was shown in Figure 2B.). We showed that both colorectal CSCs and non-CSCs were sensitized to TRAIL by miR-27a antioligonucleotides. However, we found that IC50 of TRAIL to HT29 non-CSCs reduced 39.4%, and IC50 of TRAIL to HT29 CSCs reduced 69.5% after they were transfected with miR-27a antioligonucleotides (Figure 2C). Similarly, IC50 of TRAIL to SW480 non-CSCs reduced 38.9%, and IC50 of TRAIL to SW480 CSCs reduced 64.0% after they were transfected with miR-27a antioligonucleotides (Figure 2D). These results indicated that colorectal CSCs were more sensitive to miR-27a knockdown rather than the colorectal non-CSCs when they were treated with TRAIL. We demonstrated that miR-27a antioligonucleotides resensitized colorectal cancer stem cells to TRAIL-induced cell death.

### MiR-27a antioligonucleotides enhance TRAIL-induced apoptosis in colorectal cancer stem cells

**Figure 3:**
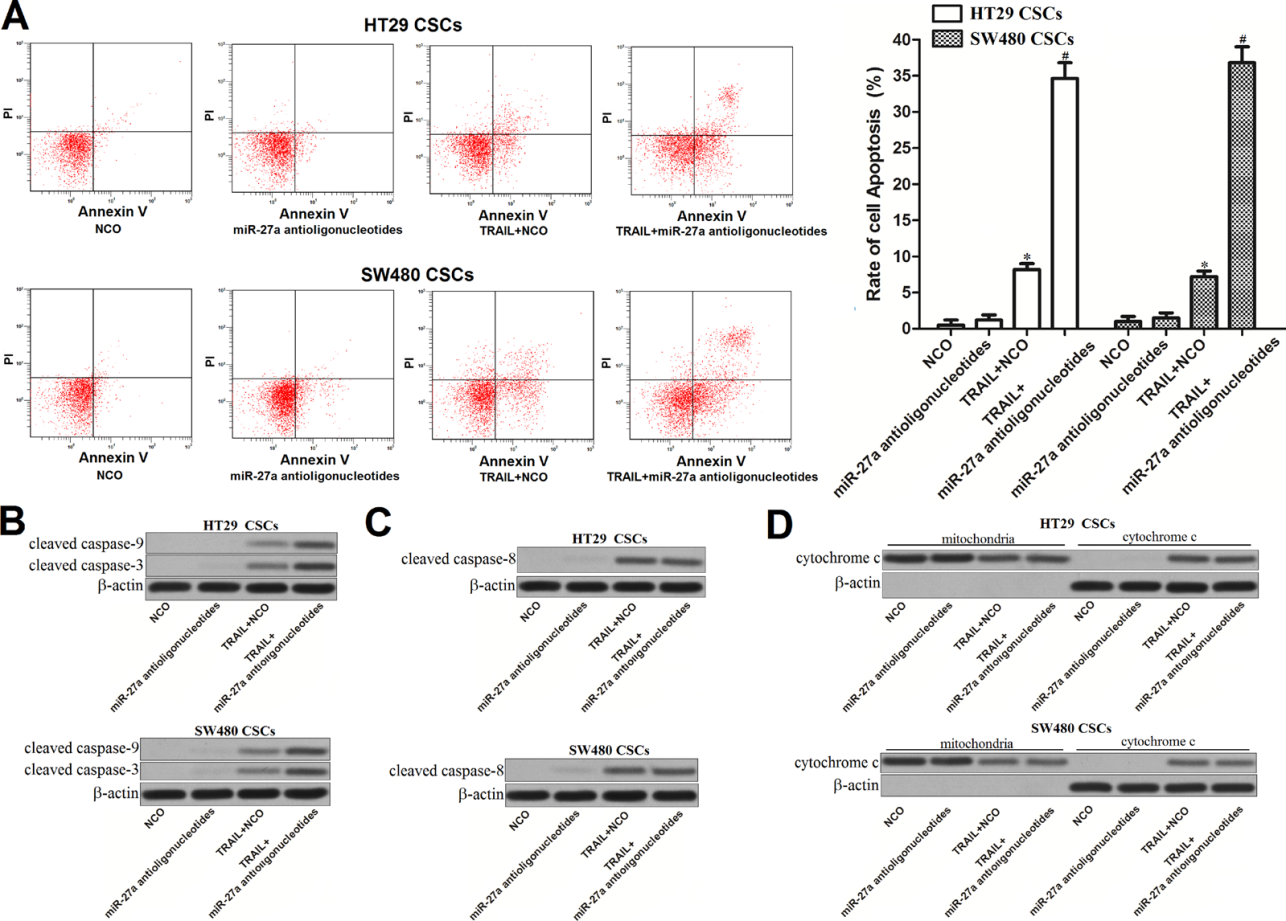
Effect of miR-27a antioligonucleotides and TRAIL on the activation of caspases and apoptosis in colorectal CSCs (**A**) HT29 and SW480 CSCs were treated with miR-27a antioligonucleotides and TRAIL (10 mg/ml). Cell apoptosis was measured by flow cytometry analysis. *P < 0.05 *vs*. NCO group, ^#^*P* < 0.05 *vs*. TRAIL + NCO group. (**B**) HT29 and SW480 CSCs were treated with miR-27a antioligonucleotides and TRAIL (10 mg/ml). Western blot analysis was performed to evaluate the activation of caspase-9 and caspase-3. (**C**) Cleavage of caspase-8 in HT29 and SW480 CSCs was detected by western blot assays. (**D**) After separation of mitochondria, cytochrome c in mitochondria and cytoplasm was detected by western blot analysis.

TRAIL is an anti-tumor drug which induces apoptosis selectively in cancer cells [13]. To investigate the role of miR-27a antioligonucleotides and TRAIL in apoptosis pathway of colorectal CSCs, flow cytometry analysis was performed. As shown in Figure 3A, although miR-27a antioligonucleotides didn't induce apoptosis directly in HT29 and SW480 CSCs, it significantly enhanced the TRAIL-induced apoptosis. Results of western blot analysis showed that miR-27a antioligonucleotides promoted the cleavage of caspase-9 and caspase-3 obviously in colorectal CSCs (Figure 3B). Interestingly, however, TRAIL-induced cleavage of caspase-8 was not influenced by knockdown of miR-27a (Figure 3C). In addition, transfection with miR-27a antioligonucleotides didn't change the release of cytochrome c in colorectal CSCs treated with TRAIL (Figure 3D).

### MiR-27a antioligonucleotides increase the expression of Apaf-1 in colorectal cancer stem cells

**Figure 4:**
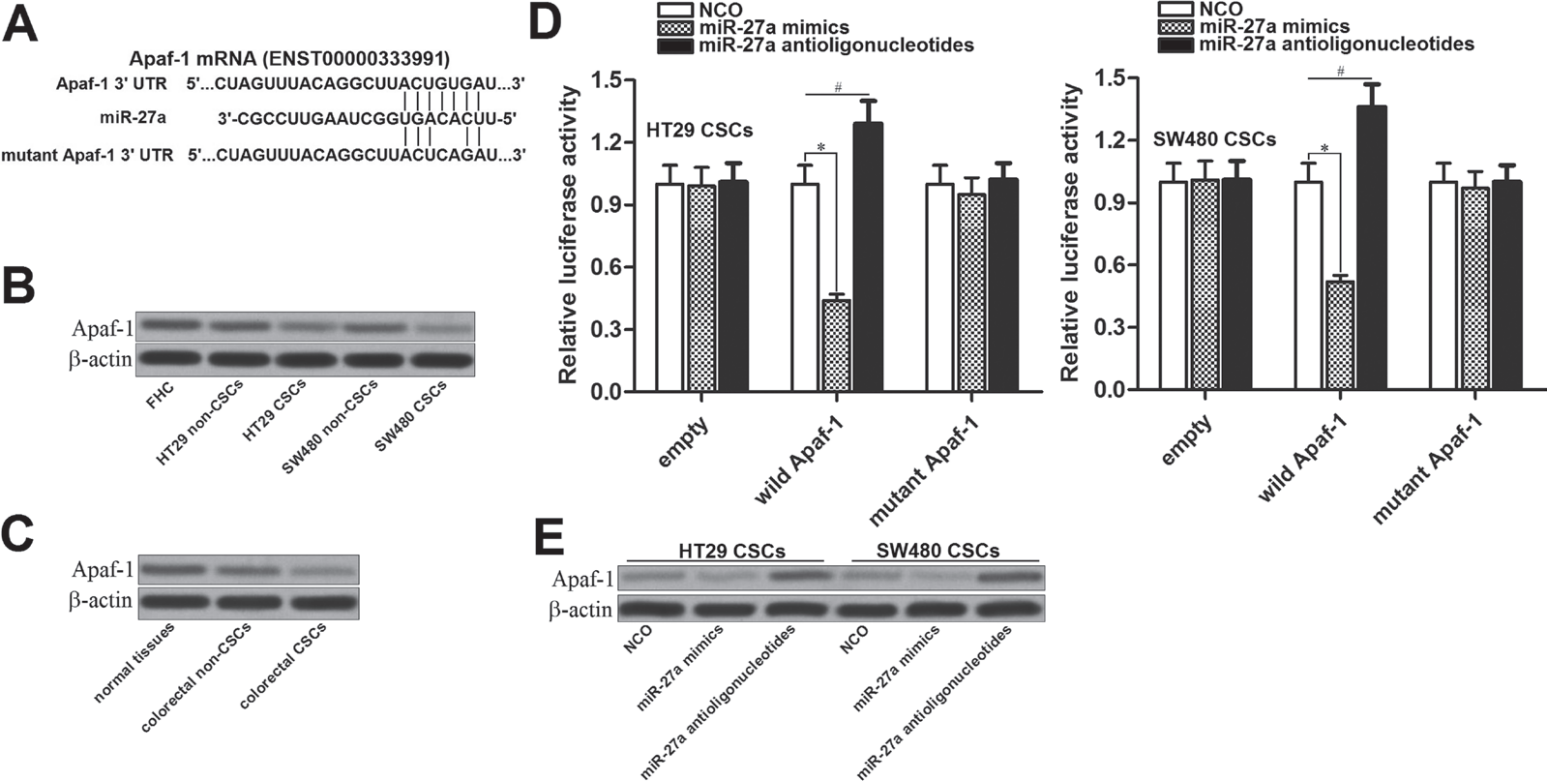
MiR-27a antioligonucleotides increase the expression of Apaf-1 in colorectal CSCs (**A**) Apaf-1 mRNA 3′ UTR contained conservative sequence at the miR-27a binding site predicted by the TargetScan database. (**B**) Expression of Apaf-1 in FHC, HT29 and SW480 CSCs and non-CSCs. (**C**) Colorectal patients’ tumor and adjacent normal tissues were digested by using collagenase type III to obtain the single cell suspension. After sorting, expression of Apaf-1 in normal colorectal tissue cells, colorectal CSCs and non-CSCs was evaluated by western blot analysis. (**D**) Luciferase reporter assay in HT29 and SW480 CSCs. The wild or mutant type of Apaf-1 3′ UTR was cloned into pMIR-REPORT plasmid and co-transfected with miR-27a mimics or antioligonucleotides. Dual-Luciferase Reporter Assay System was used to detect the luciferase activities. *P < 0.05. ^#^*P* < 0.05. (**E**) Effect of miR-27a mimics and antioligonucleotides on changing the expression of Apaf-1 in HT29 CSCs and non-CSCs.

To explore the mechanism by which miR-27a antioligonucleotides facilitated TRAIL-induced apoptosis in colorectal CSCs, we searched the target gene of miR-27a on the public microRNA database of TargetScan (http://www.targetscan.org/). Apoptotic protease activating factor-1 (Apaf-1), which was identified as pro-apoptotic gene [21], contained conservative 3′ UTR sequence at the miR-27a binding site (Figure 4A). In addition, results of western blot analysis showed that expression of Apaf-1 was decreased in colorectal CSCs compared to their corresponding non-CSCs (Figure 4B and 4C). Because of the negative correlation between the expression of miR-27a and Apaf-1, we inferred that Apaf-1 gene was the target of miR-27a in colorectal CSCs. Luciferase assays revealed that miR-27a mimics suppressed reporter gene activity in both HT29 CSCs and SW480 CSCs. On contrary, miR-27a antioligonucleotides increased the reporter gene activity in them (Figure 4D). Furthermore, after transfection with miR-27a antioligonucleotides, the protein level of Apaf-1 was significantly increased in both HT29 and SW480 CSCs (Figure 4E). These results demonstrated that the Apaf-1 gene was the target of miR-27a, knockdown of which increased the expression of Apaf-1 in colorectal CSCs.

### MiR-27a antioligonucleotides enhanced the anti-tumor effect of TRAIL on colorectal cancer stem cells via increasing the expression of Apaf-1

**Figure 5:**
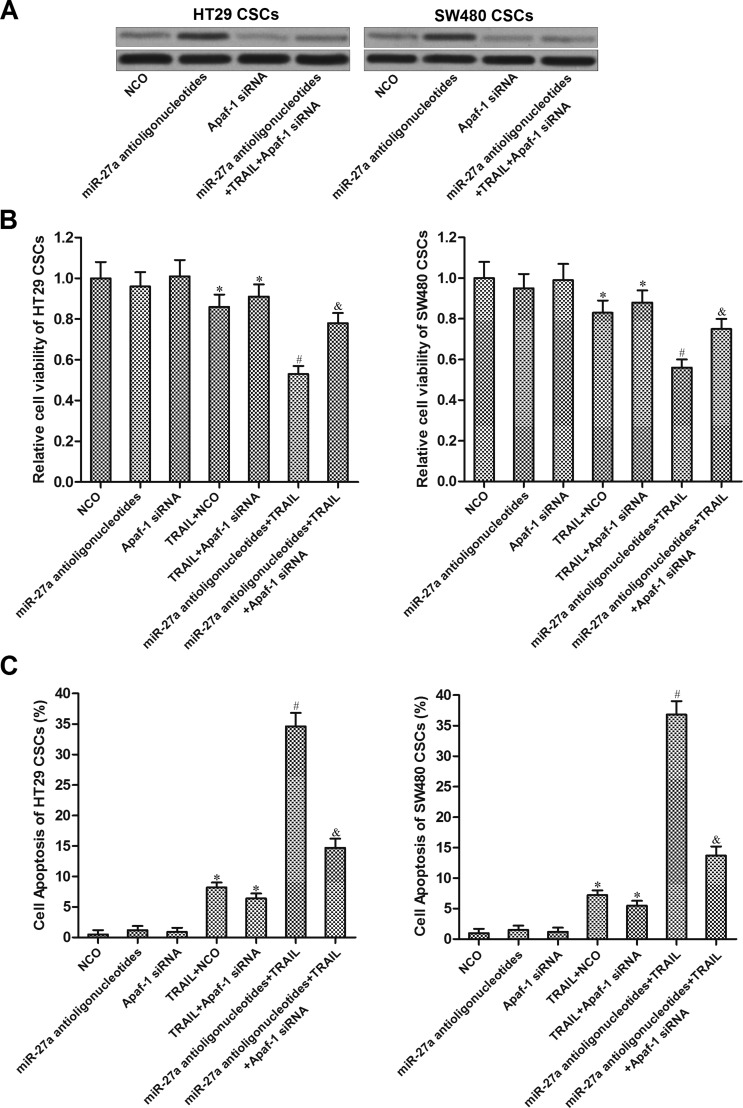
MiR-27a antioligonucleotides sensitized colorectal CSCs to TRAIL by increasing the expression of Apaf-1 (**A**) Effect of miR-27a antioligonucleotides and Apaf-1 siRNA on changing the expression of Apaf-1 was evaluated by western blot analysis. (**B**) HT29 and SW480 CSCs were treated with NCO, miR-27a antioligonucleotides, Apaf-1 siRNA and TRAIL (10 mg/ml). Cell viability was measured by using CCK-8 assays. *P < 0.05 *vs*. NCO group, ^#^*P* < 0.05 *vs*. TRAIL + NCO group, ^&^*P* < 0.05 *vs*. miR-27a antioligonucleotides + TRAIL group. (**C**) HT29 and SW480 CSCs were treated with NCO, miR-27a antioligonucleotides, Apaf-1 siRNA and TRAIL (10 mg/ml). Cell apoptosis was measured by flow cytometry. *P < 0.05 *vs*. NCO group, ^#^*P* < 0.05 *vs*. TRAIL + NCO group, ^&^*P* < 0.05 *vs*. miR-27a antioligonucleotides + TRAIL group.

To investigate whether miR-27a antioligonucleotides sensitized colorectal CSCs to TRAIL by increasing the expression level of Apaf-1, we knockdown Apaf-1 gene by using its specific siRNA. Effect of Apaf-1 siRNA and miR-27a antioligonucleotides on changing the expression of Apaf-1 was shown in Figure 5A. Results of cell viability assays showed that transfection with miR-27a antioligonucleotides significantly enhanced the anti-tumor effect of TRAIL on HT29 and SW480 CSCs. However, the activity of miR-27a antioligonucleotides was inhibited by Apaf-1 siRNA. Knockdown of Apaf-1 abolished the promotion of miR-27a antioligonucleotides on TRAIL-induced cell death (Figure 5B). Similarly, although miR-27a antioligonucleotides increased the apoptotic rate of HT29 and SW480 CSCs under the treatment of TRAIL, its activity was significantly suppressed by transfection with Apaf-1 siRNA (Figure 5C). These results indicated that miR-27a antioligonucleotides sensitized colorectal cancer stem cells to TRAIL by increasing the expression of Apaf-1.

### MiR-27a antioligonucleotides promote the formation of Apaf-1-caspase-9 complex in TRAIL-treated colorectal cancer stem cells

**Figure 6:**
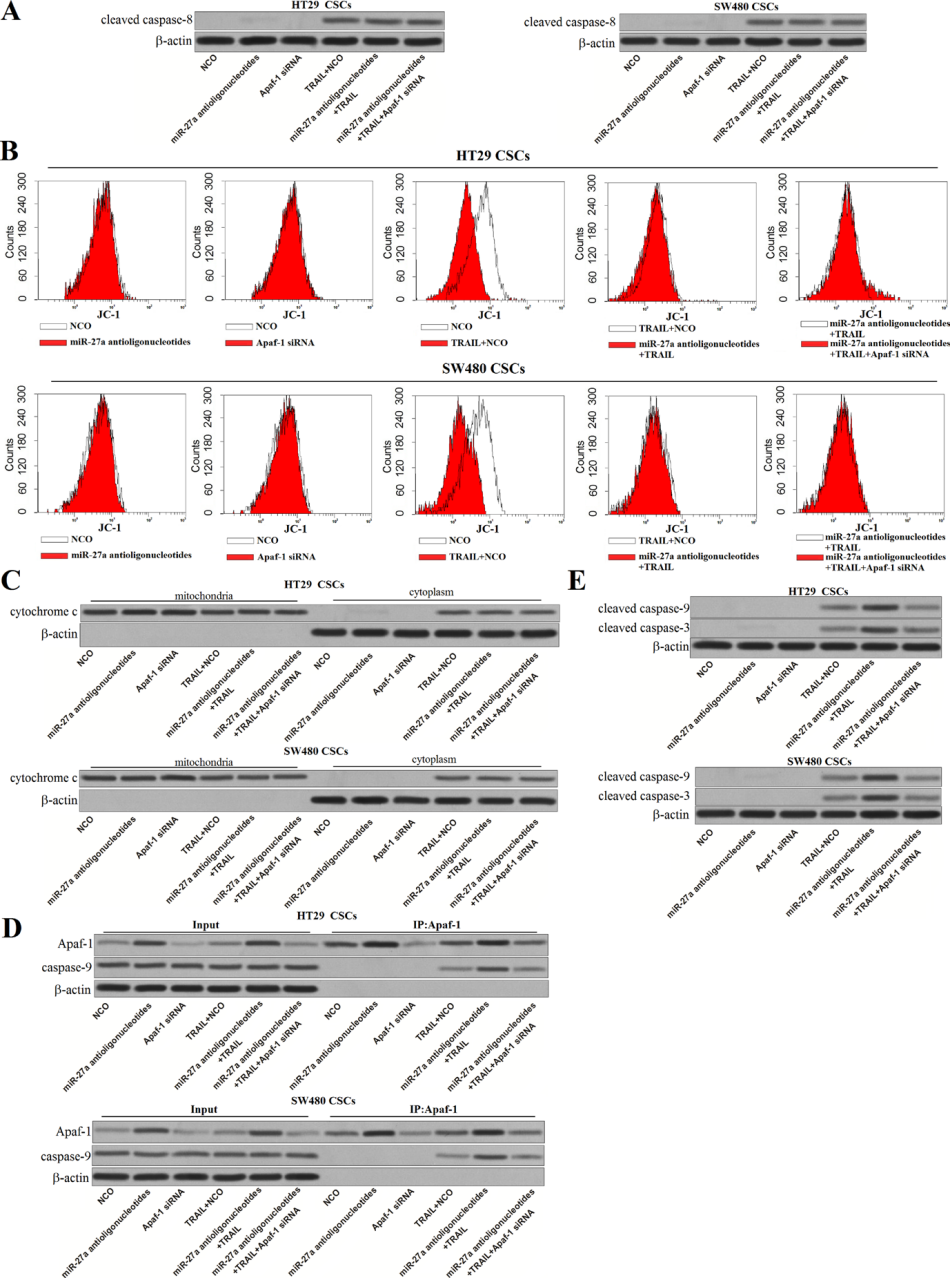
MiR-27a antioligonucleotides promote the formation of Apaf-1-caspase-9 complex in TRAIL-treated colorectal CSCs (**A**) After treatment with miR-27a antioligonucleotides, Apaf-1 siRNA and TRAIL, cleavage of caspase-8 was evaluated by western blot analysis in HT29 and SW480 CSCs. (**B**) Mitochondrial membrane potential (∆ Ψ_m_) was detected by using JC-1 on flow cytometry. (**C**) After separation of mitochondria, cytochrome c in mitochondria and cytoplasm was detected by western blot analysis. (**D**) Co-immunoprecipitation and western blot analysis was performed to evaluate the formation of Apaf-1-caspase-9 complex. (**E**) Cleavage of caspase-9 and caspase-3 in HT29 and SW480 CSCs was evaluated by western blot analysis.

Caspase-8 is the substrate of TRAIL-death receptor complex [21]. However, results of western blot analysis showed that transfection with miR-27a antioligonucleotides didn't influence the activation of caspase-8 induced by TRAIL. Besides, Apaf-1 siRNA, which inhibited the overexpression of Apaf-1 induced by miR-27a antioligonucleotides, had no effect on the cleavage of caspase-8 in colorectal CSCs (Figure 6A). As the downstream of TRAIL/caspase-8 signaling, TRAIL treatment induced collapse of mitochondrial membrane potential (∆ Ψ_m_). However, transfection with MiR-27a antioligonucleotides as well as Apaf-1 siRNA didn't change the effect of TRAIL on (∆ Ψ_m_) in colorectal CSCs (Figure 6B). In addition, as the results of ∆ Ψ_m_ decrease, TRAIL but not the miR-27a antioligonucleotides and Apaf-1 siRNA, induced release of cytochrome c in HT29 and SW480 CSCs (Figure 6C). In the presence of cytochrome c, Apaf-1 conjugated to pro-caspase-9 and triggered it [22]. To investigate the role of miR-27a antioligonucleotides and TRAIL in the formation of Apaf-1-caspase-9 complex, co-immunoprecipitation assay was performed. We found that TRAIL treatment induced interaction with Apaf-1 and caspase-9. Furthermore, although miR-27a antioligonucleotides single treatment didn't induce the formation of Apaf-1-caspase-9 complex, it significantly enhanced the effect of TRAIL on formatting this complex. However, in case of Apaf-1 was transfected into colorectal CSCs, interaction with Apaf-1 and caspase-9 induced by combination with miR-27a antioligonucleotides and TRAIL was inhibited (Figure 6D). We therefore demonstrated that miR-27a antioligonucleotides promoted the formation of Apaf-1-caspase-9 complex by increasing the expression of Apaf-1 in TRAIL-treated colorectal CSCs. In addition, as the results of Apaf-1-caspase-9 complex formation, caspase-9 and its substrate caspase-3 was cleaved and activated [23]. Consistent with this, results of western blot showed that miR-27a antioligonucleotides significantly enhanced the TRAIL-induced activation of caspase-9 and caspase-3 (Figure 6E), which finally induced apoptosis. Taken together, our results demonstrated that miR-27a antioligonucleotides promoted TRAIL-induced apoptosis in colorectal CSCs by increasing the expression of Apaf-1, which conjugated to caspase-9 and triggered it.

## DISCUSSION

As microRNAs are important regulators in multiple cellular processes, cancer cells, especially the cancer stem cells, usually change the expression profile of microRNAs to survive and proliferate rapidly [24]. In addition, studies have demonstrated that drug-resistance of CSCs is usually induced by dysregulation of microRNAs. Correcting the expression profile of microRNAs is reported to resensitize the CSCs to treatment of anti-tumor drugs [25, 26].

MicroRNA-27a (miR-27a) has been reported to function as an oncogene in multiple cancers. Overexpression of miR-27a was found to markedly promote cell proliferation, migration, invasion and epithelial-mesenchymal transition in different cancers including colorectal cancer [27–30]. Furthermore, previous researches demonstrated that increased expression of miR-27a is involved in resistance to chemotherapy in cancers [31, 32]. In this study, we demonstrate that miR-27a is overexpressed in colorectal cancer, especially in the CD133 positive stem-like cell population of colorectal cancer (colorectal CSCs). Interestingly, although the colorectal CSCs showed obvious resistance to TRAIL treatment, knockdown of miR-27a by its specific antioligonucleotides resensitized them to TRAIL.

TRAIL has been considered as a promising new therapeutic drug for cancers, which induces death of cancers by triggering apoptosis pathway [33]. After administration with TRAIL, it binds to death receptors 4/5 (DR4/5) to form a death-inducing signaling complex (DISC). DISC then recruits pro-caspase-8 and triggers it. Subsequently, cleaved caspase-8 decreases the potential of mitochondrial membrane and induces the formation of mitochondrial membrane pore. Cytochrome c, which is recognized as an apoptotic inducer, is then released into cytoplasm from the damaged mitochondria [34–37]. In the presence of cytochrome c and dATP, pro-caspase-9 is recruited to Apaf-1 to form the Apaf-1/caspase-9 apoptosome. As the results, caspase-9 and its substrate of caspase-3 are activated, and finally, apoptosis occurs [38].

**Figure 7:**
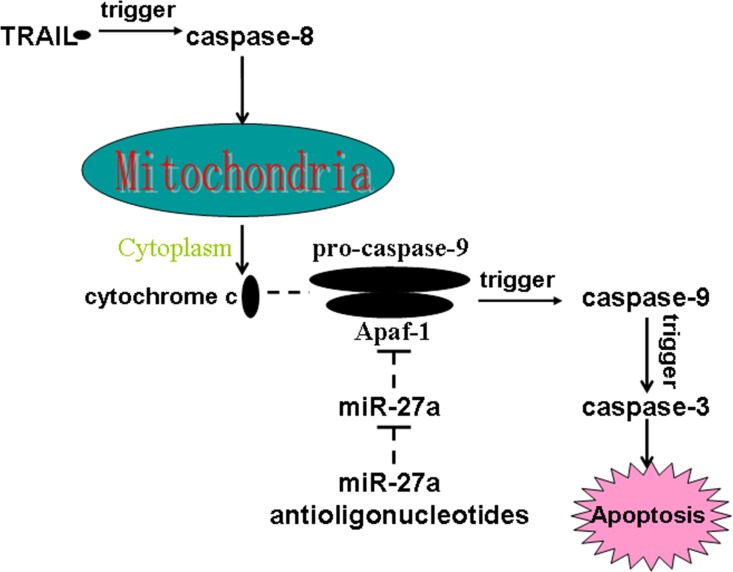
Schema of the mechanisms implicated in miR-27a antioligonucleotides-sensitized apoptosis in TRAIL-treated colorectal CSCs MiR-27a antioligonucleotides promote TRAIL-dependent formation of Apaf-1/caspase-9 complex by increasing the expression of Apaf-1. As a result, caspase-9 is triggered, followed by activation of caspase-3 and occurrence of apoptosis finally.

In the present study, we proved that miR-27a antioligonucleotides resensitized colorectal cancer stem cells to TRAIL-induced apoptosis. However, miR-27a antioligonucleotides treatment didn't promote the activation of caspase-8 and release of cytochrome c in TRAIL-treated colorectal cancer stem cells. Mechanically, our results demonstrated that miR-27a antioligonucleotides increased the expression of Apaf-1 in TRAIL-treated colorectal cancer stem cells. Therefore, miR-27a antioligonucleotides increased the level of Apaf-1/caspase-9 apoptosome, which caused the activation of caspase-9 and subsequent activation of caspase-3. Schema of the mechanisms implicated in miR-27a antioligonucleotides-sensitized apoptosis in TRAIL-treated colorectal cancer stem cells is shown in Figure 7.

Drug-resistance is a major obstacle for clinical use of TRAIL treatment [39]. Here, we provide evidences that miR-27a antioligonucleotides sensitize colorectal cancer stem cells to TRAIL by promoting the formation of Apaf-1-caspase-9 complex. Knockdown of miR-27a may represent a novel therapeutic strategy for overcoming the resistance of cancer stem cells to TRAIL.

## MATERIALS AND METHODS

### Tissue samples

A total of 25 primary colorectal cancer tissues and their corresponding non-tumor normal tissues were obtained from patients who underwent tumor resection in Cancer Hospital of China Medical University from 2/2014 to 10/2016. Tumor tissues were digested by using collagenase type III (Worthington Biochemical, USA) to obtain the single cell suspension. The tumor specimens used in the present study were obtained with the approval of the ethics committee of Cancer Hospital of China Medical University, and all of the patients had given their informed consent.

### Cell lines

Colorectal cancer cells lines HT29 and SW480 were originated from American Type Culture Collection (ATCC, USA). Primary cultures of human normal colorectal cell line FHC was purchased from ATCC. Cells were grown in Dulbecco's modified Eagle's medium (DMEM, Invitrogen, USA) supplemented with 10 % fetal bovine serum (FBS) in a 37°C humidified chamber with 5% CO_2_.

### Analysis of CD133 by flow cytometry and cell sorting

CD133-FITC antibody (BD Pharmingen, USA) was used for identifying CSCs in colorectal cancer cells. Briefly, single cell suspension obtained from primary colorectal cancer patients, HT29 and SW480 cells were incubated with CD133-FITC antibody on ice for 40 min in the dark. After incubation, these cells were analyzed on FACSAria (BectonDickinson Biosciences) flow cytometer. CD133 positive HT29 and SW480 cells were considered as the colorectal CSCs for sorting.

### Quantitative real-time polymerase chain reaction (qRT-PCR)

Total RNA was extracted from cultured HT29 and SW480 cells and colorectal cancer patients’ tumor tissues by using TRIzol reagent (Invitrogen, USA). One Step PrimeScript miRNA cDNA Synthesis Kit (TaKaRa, China) was used to synthesize the cDNA for miR-27a. The qRT-PCR reactions for miR-27a were performed by using the SYBR Premix Ex Taq (TaKaRa) on Applied Biosystems 7500 Sequence Detection system (Applied Biosystems, USA). The relative expression of miR-27a was normalized by U6 snRNA expression.

### Transient transfection with RNAs

Negative control oligonucleotides (NCO), miR-27a mimics, miR-27a antioligonucleotides were purchased from RiboBio Co. Ltd. (China). Apaf-1 siRNA was purchased from Cruz Biotechnology, Inc (USA). These RNAs (50 pmol/ml) were transient transfected into HT29 CSCs (non-CSCs) and SW480 CSCs (non-CSCs) by using lipofectamine 2000 (Invitrogen) according to the manufacturer's guidance.

### Luciferase reporter assays

To verify the miR-27a-targeted mRNA, wildtype 3′ UTR of Apaf-1 mRNA (ENST00000333991) was PCR-amplified and inserted into the pMIR-REPORT luciferase reporter vector (Ambion, USA). Site-Directed Mutagenesis Kit (TaKaRa) was used to create the pMIR-REPORT luciferase reporter contained mutant sequence of Apaf-1 3′ UTR at the complementary binding site of miR-27a. Luciferase reporter assays were performed by using dual-luciferase reporter assay system (Promega, USA) according to the manufacturer's instruction. pRL-TK vector (Promega) which expresses Renilla luciferase) was used as an internal control.

### Detection of cytochrome c release

To separate mitochondria from cytoplasm in HT29 and SW480 CSCs, mitochondria/Cytosol Fraction Kit (BioVision, USA) was used according to the manufacturer's protocol. Protein levels of cytochrome c in mitochondria and cytoplasm were detected to evaluate the release of cytochrome c from mitochondria into cytoplasm.

### Cell viability assays

Colorectal cancer cells were seeded in 96-well plates at a density of 5 × 10^3^ cells/well overnight. Cells were then transfected with RNA oligonucleotides and treated with different concentrations of TRAIL subsequently. After treatment, CCK-8 detection kit (Sigma-Aldrich, USA) was used to measure cell viability according to the manufacturer's protocol. Briefly, 20 μl/well CCK-8 was added to the medium and incubated at 37°C for 2 h. The absorbance at 450 nm was measured by a microplate reader to determine viable cells. Half maximal inhibitory concentration (IC50) of TRAIL to colorectal cancer cells was calculated according to the cell viability curves conducted by CCK-8 assays.

### Co-immunoprecipitation

Cells were harvested and incubated with lysis buffer (Cell Signaling Technologies, USA) for 10 min on ice. Cellular debris was removed by centrifugation for 5 min at 12,000 g to collect the supernatant. Protein lysates were incubated with primary antibody of Apaf-1 (Cell Signaling Technologies) overnight at 4°C, followed by followed by addition of protein G agarose beads for 2 h at 4°C. Immunoprecipitates were washed with cold lysis buffer three times, and subsequently boiled with sodium dodecyl sulfate (SDS) sample buffer.

### Western blot

Proteins were extracted from cells by using Cell Signaling lysis buffer. Subsequently, the proteins were boiled with SDS sample buffer and separated by sodium dodecyl sulfate polyacrylamide gel electrophoresis (SDS-PAGE). The separated proteins were then transferred to PVDF membranes (Millipore, USA). After trarsmembrane, membranes contained proteins were incubated with the primary antibodies overnight at 4°C. After incubation with horseradish peroxidase-conjugated second antibody (Cell Signaling Technology), signals were detected using enhanced chemiluminescence reagents (Thermo, USA).

### Detection of apoptosis

Cells were incubated with Annexin-V-FITC and propidium iodide (PI) following the instruction of apoptosis detecting kit (Invitrogen). Apoptosis of colorectal cancer cells was detected on flow cytometry (BectonDickinson Biosciences).

### Measurement of mitochondrial membrane potential (∆ Ψ_m_)

Mitochondrial membrane potential was measured to evaluate the damage of mitochondria. Cells were incubated with 5,5′,6,6′-Tetrachloro-1,1′,3,3′-tetraethyl imidacarbo cyanine iodide (JC-1, Molecular Probes, USA). Red fluorescence was emitted by JC-1 polymer in healthy mitochondria and detected by flow cytometry.

### Statistical analysis

All values were depicted as mean ± standard deviation and carried out by three independent experiments. All data were statistically analyzed by Student's *t*-test or one-way ANOVA with a Bonferroni correction using SPSS 15.0 software. Values of *P* < 0.05 were considered significant.
